# 3D Cultures of Prostate Cancer Cells Cultured in a Novel High-Throughput Culture Platform Are More Resistant to Chemotherapeutics Compared to Cells Cultured in Monolayer

**DOI:** 10.1371/journal.pone.0111029

**Published:** 2014-11-07

**Authors:** Karen F. Chambers, Eman M. O. Mosaad, Pamela J. Russell, Judith A. Clements, Michael R. Doran

**Affiliations:** 1 Stem Cell Therapies Laboratory, Queensland University of Technology at the Translational Research Institute, Brisbane, Queensland, Australia; 2 Institute of Health and Biomedical Innovation, Queensland University of Technology, Brisbane, Queensland, Australia; 3 Australian Prostate Cancer Research Centre, Translational Research Institute, Brisbane, Queensland, Australia; 4 Mater Research Institute, The University of Queensland, Translational Research Institute, Brisbane, Queensland, Australia; Roswell Park Cancer Institute, United States of America

## Abstract

Despite monolayer cultures being widely used for cancer drug development and testing, 2D cultures tend to be hypersensitive to chemotherapy and are relatively poor predictors of whether a drug will provide clinical benefit. Whilst generally more complicated, three dimensional (3D) culture systems often better recapitulate true cancer architecture and provide a more accurate drug response. As a step towards making 3D cancer cultures more accessible, we have developed a microwell platform and surface modification protocol to enable high throughput manufacture of 3D cancer aggregates. Herein we use this novel system to characterize prostate cancer cell microaggregates, including growth kinetics and drug sensitivity. Our results indicate that prostate cancer cells are viable in this system, however some non-cancerous prostate cell lines are not. This system allows us to consistently control for the presence or absence of an apoptotic core in the 3D cancer microaggregates. Similar to tumor tissues, the 3D microaggregates display poor polarity. Critically the response of 3D microaggregates to the chemotherapeutic drug, docetaxel, is more consistent with *in vivo* results than the equivalent 2D controls. Cumulatively, our results demonstrate that these prostate cancer microaggregates better recapitulate the morphology of prostate tumors compared to 2D and can be used for high-throughput drug testing.

## Introduction

Three-dimensional (3D) cell culture is motivated by the need to carry out experiments that better recapitulate the physiological microenvironment. Conventional two dimensional (2D) cell cultures often fail to mimic the cellular functions and signaling pathways present in tissue. Consequently 2D cell cultures can lead to skewed and limited data [1], [2]. Microarray profiling of 2D versus 3D cultures has shown that 50% of genes change in expression upon 3D culture [3]. Some of these differences may be attributed to differences in the mechanical tension of the matrix. For instance, cells cultured in 2D on tissue culture plastic experience elevated tensile stress, a million times greater than that of soft tissue [4], and this is known to alter cell physiology [5]. Artificially high tensile stresses can profoundly affect cell morphology, cytoskeleton arrangement, cell-cell adhesion and migration. 3D-cultures better mimic natural tissue mechanical stresses and thus provide a more representative pathophysiological condition than using conventional tissue culture plates [6], [7]. This relative effect is apparent during *in vitro* chemotherapy testing, where 2D cultures are typically hypersensitive to drugs whilst 3D culture drug sensitivity more often parallels the equivalent *in vivo* scenario [8], [9].

Despite the fact that 3D-cultures function as more robust *in vitro* cancer drug testing models, most laboratories still rely on 2D cultures as their primary tool. This is partly due to the increased labor and costs associated with establishing 3D models and because there is no agreement on a single standard model which could be used across the field. Several types of 3D culture systems, with different advantages and pitfalls, are currently employed. Natural extra-cellular matrix (ECM) gels such as type-I collagen and laminin-rich Matrigel can provide the mechanical and chemical cues for tissue morphogenesis, however, they contain a number of undefined growth factors and ECM proteins that differ between batches changing the mechanical properties of the gel. Synthetic gels comprised of peptide-functionalized synthetic polymers are tailored to mimic specific ECM properties and therefore offer an alternative [10]. However, scaffold and gel based systems can be expensive to scale up into large high-throughput studies and difficult to analyze. Techniques such as liquid-agar overlay and polyhema offer a cheaper alternative, however the size and uniformity of the aggregates cannot be strictly regulated and this would translate to different drug penetration rates [11], [12]. We have adapted a high throughput microwell system to culture prostate cells as microaggregates of a controlled size. This system offers an advantage over other 3D culture systems in that the dimensions of the microaggregates can be strictly regulated and aggregates of a defined size are produced of a scalable nature for high-throughput drug testing. Herein we show that prostate cancer cells self-assemble into aggregates that respond to drug treatment in a manner consistent with the expected *in vivo* sensitivity.

## Materials and Methods

### Fabrication and multi-layering of the microwells

**Figure 1:**
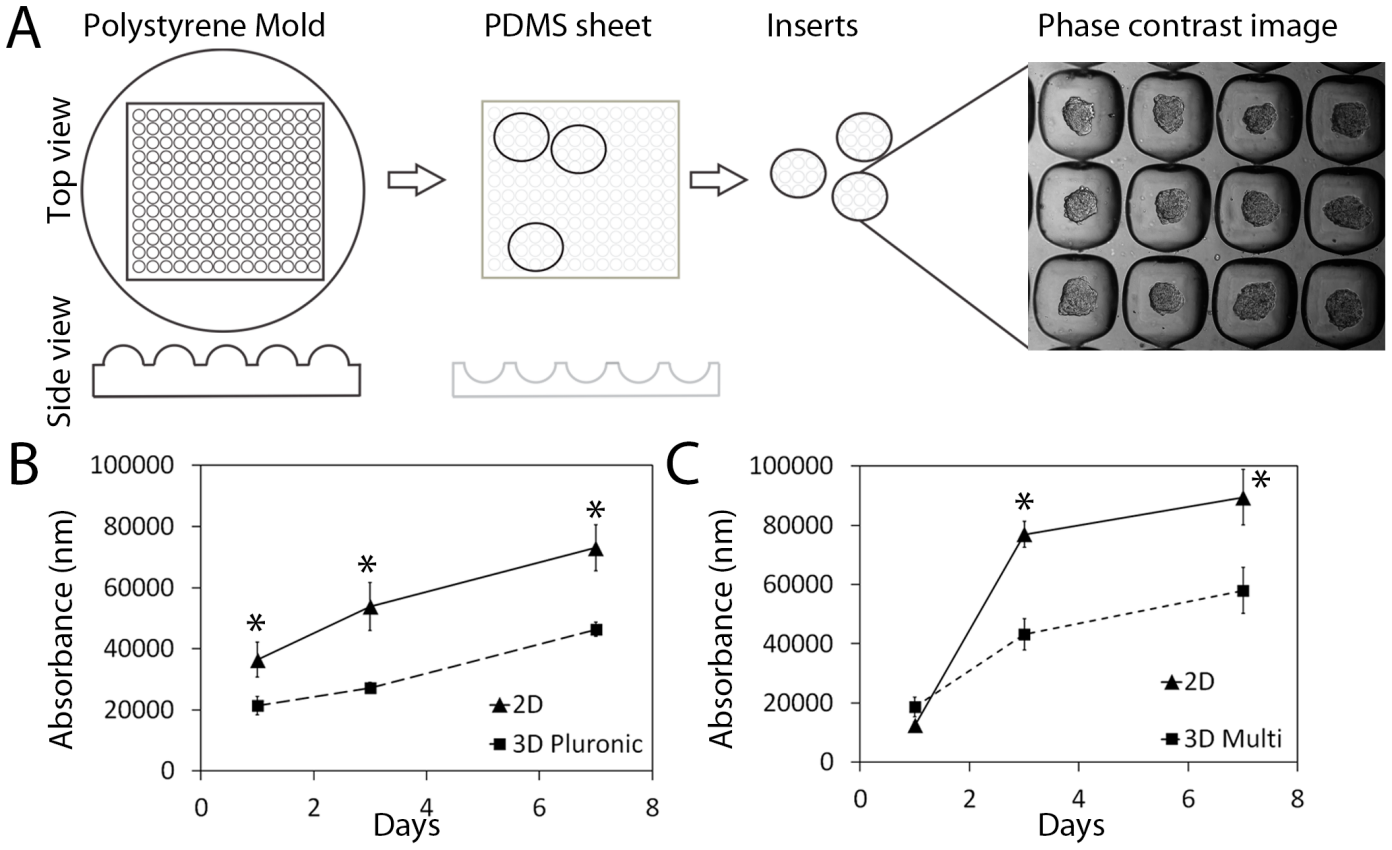
Fabrication of the PDMS micro-patterned surface system and surface coating with pluronic or multi-layering. (A) A polystyrene mold [13], was used to cast PDMS sheets with a micro-patterned surface. Discs were punched out of the PDMS sheet to form inserts for multiwell plates, and these surfaces were coated with either 5% pluronic or multilayered with CHI and HA. LNCaP cells (50,000) were seeded on to either (B) pluronic coated (3D pluronic) or (C) multi-layered (3D multi) microwells and the Alamar blue assay was used to measure metabolism in 3D versus 2D cells. Both coatings produced viable aggregates that increased in metabolism over 7 days at a comparable rate to cells grown in 2D culture. Three technical replicates were carried out per time-point.

The fabrication of the polydimethylsiloxane (PDMS) microwell arrays was performed as described previously [13]. In this instance we used soft lithography to form arrays of 360×360×180 µm microwells or 800×800×800 µm on PDMS discs, which were then mounted into the wells of a 48-well tissue culture plate. Briefly, a silica wafer was used to form a PDMS mold, from which an inverse polystyrene mold was created. The PDMS microwell surface was created in sheets using the latter mold and the sheet was punched out into discs (**Figure 1A**). Punches of differing sizes can be used to create inserts for any sized tissue culture plastic vessel. Using this technique thousands of microwells can be produced *en masse* (600 microaggregates/cm^2^ or 150 microaggregates/cm^2^ for the smaller and larger microwells, respectively). The PDMS surface was either coated with 5% pluronic/phosphate buffered saline (PBS) solution or multilayered with chitosan (CHI) and hyaluronic acid (HA), both of which prevent cell adhesion to the PDMS surface. As described previously [13] multi-layering begins with an electropositive poly-lysine layer to aid further layer adhesion and five layers of CHI and HA are sequentially incubated for 15 minute intervals. We have previously shown that the upper HA layer blocks cell adhesion on PDMS microwells and that this promotes cell aggregation [14]. After coating the inserts were sterilized in 70% ethanol and washed overnight with PBS ready for use.

### Cells and prostate microaggregate culture

The prostate cancer cell lines, RWPE-2 [15] and LNCaP [16], and non-cancerous cells line RWPE-1 [15], were obtained from the American Type Culture Collection (ATCC). Cells were cultured in RPMI 1640, 5% fetal bovine serum (FBS) (Gibco) plus 1% penicillin/streptomycin (P/S)(Gibco) in 5% CO_2_ at 37°C. For medium optimization studies, Keratinocyte-SFM, 2% Bovine Pituitary Extract (BPE), plus epidermal growth factor (EGF) supplement (Gibco) was also used. Either 50,000 or 100,000 cells were seeded per well in 1 mL of culture medium. Microaggregates were formed through forced aggregation; the plates were centrifuged at 1000 rpm for 5 min to facilitate pelleting of the cells into the microwells. In the forced aggregation process, the cells suspended in the medium are uniformly pelleted within the array of microwells at the base of the well. Medium was exchanged on days 3, 6, 8, 10, and 13. During the medium exchange, care was taken not to aspirate the microaggregates. Medium was added and subtracted from the same point in the well during each exchange. Plates were incubated at 37°C with 5% CO_2_. Phase contrast images were taken using a Nikon Eclipse-Ti inverted microscope and the central diameter of each microaggregate was measured using Image-J software (http://rsbweb.nih.gov/ij/). A minimum of 50 microaggregates were measured per time-point.

### Sectioning, immunofluorescence and confocal imaging

Microaggregates were fixed with 4% paraformaldehyde (PFA) for 30 mins and either embedded in Tissue-Tek optimal cutting temperature (OCT) compound (VWR International) for sectioning or immunostained directly. Samples were permeabilized with 0.5% Triton-X 100 for 20 min and incubated with primary antibodies for E-Cadherin (Invitrogen, AB_86564), β-catenin (Santa-Cruz, AB_626807) (diluted 1∶200), α6-integrin (Millipore, AB_10834933), laminin-5 (Abcam, AB_2133782), cleaved caspase-3 (Cell Signalling Technology, AB_331440) (diluted 1∶100) and Ki67 (Abcam, AB_443209) (1∶400) overnight at 4°C. The respective secondary antibody conjugated to alexa-488 (Invitrogen) was added for 1 hr at room temperature; 4',6-diamidino-2-phenylindole (DAPI, Sigma-Aldrich) was used to stain the nuclei and Rhodamine Phalloidin (Invitrogen) was used to stain for F-actin. The microaggregates were mounted using Prolong gold (Invitrogen) and imaged using a Leica TCS SP5 confocal microscope or a Nikon Eclipse-Ti inverted microscope.

### Live/dead staining

Cells were incubated with 2 µg/mL fluorescein diacetate (FDA) (Sigma-Aldrich) for 15 mins and 20 µg/mL propidium iodide (PI) (Sigma-Aldrich) for 2 mins at 37°C to stain live and dead cells respectively. FDA is a cell-permeable esterase substrate that measures both enzymatic activity and cell-membrane integrity. Propidium iodide binds to DNA, but is only able to penetrate the compromised membranes of dead or dying cells. Cells were imaged using a Leica TCS SP5 confocal microscope and the percentage area of dead cells per microaggregate was quantified using Image-J software (http://rsbweb.nih.gov/ij/). A minimum of 10 microaggregates were analyzed per time-point.

### Alamar blue and WST-1 cell viability assays

To establish cell metabolism over 14 days of microaggregate growth, the Alamar blue (Invitrogen) assay was used as per the manufacturer's instructions. On days 1, 3, 7 and 14 a 3% volume of Alamar blue was added per well. The plates were incubated at 37°C for 1 hr and 100 µl medium transferred to a 96-well black plate. Fluorescence (excitation 544 nm, emission 590 nm) was detected using a plate reader (BMG Omega, BMG LABTECH). WST-1 (Roche) was used to quantify the viability of RWPE-1 and RWPE-2 cells cultured in different medium types. Cells were cultured for 3 days followed by the addition of 10% WST-1 for 1 hr before reading absorbance at 450 nm on a Bio-Rad Benchmark Plus plate reader.

#### PicoGreen assay

The PicoGreen assay (Invitrogen) used as a measure of viable cells by detecting total double stranded DNA, was performed according to the manufacturer's instructions. Briefly, DNA was extracted with 0.5 mg/mL proteinase K (Roche) phosphate buffered EDTA (PBE). Samples were diluted (1∶10) in RNaseA solution (Invitrogen) and incubated with PicoGreen assay reagent before being read on a fluorescence plate reader (BMG Omega, BMG LABTECH; excitation 485 nm emission 520 nm).

### Drug treatments

LNCaP cells were seeded in 48-well plates with or without PDMS inserts and cultured for two days prior to treatment with dilutions of 0.01–1000 nM docetaxel (Sigma-Aldrich) for 72 h, which were compared to a DMSO vehicle control. The protocol for Alamar blue was adapted to read within the plate through the clear PDMS insert by using 10% Alamar blue and incubating for 2 hrs. A serial dilution of cells showed that the fluorescence was relative to cell number (R-squared value of 0.99; data not shown). The IC50 was calculated using Calcusyn Statistics package (Biosoft, UK) as reported previously [17].

### 3D culture of RWPE-1 cells in Matrigel basement membrane matrix

RWPE-1 cells were cultured in Matrigel (BD Biosciences) for 7 days to assess polarity. This method has been used previously [18], [19]; briefly, RWPE-1 cells were seeded in either 1% or 8% Matrigel in the presence of Keratinocyte-SFM medium supplemented with 2% BPE (Gibco). The polarity of cells grown in Matrigel plus or minus microwell insert was compared.

## Results and Discussion

### The PDMS microwell system produces prostate cancer microaggregates of uniform and controlled size

**Figure 2:**
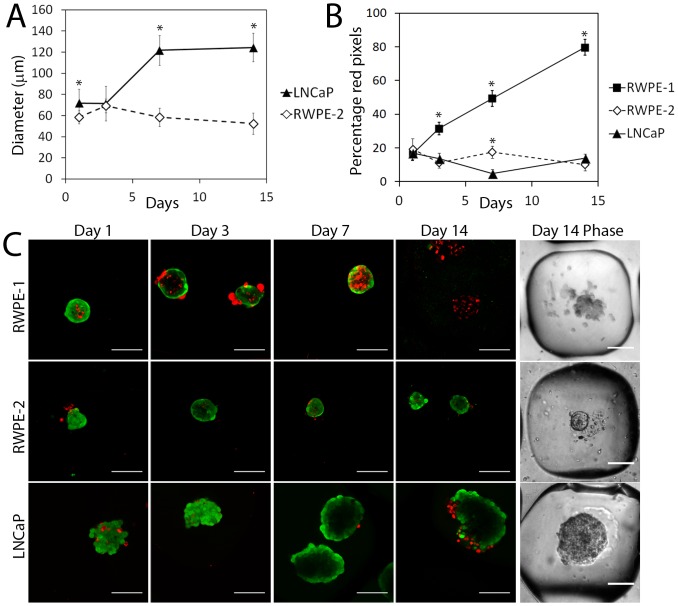
The PDMS microwell system produces prostate cancer cell aggregates that are viable and of a controlled size. (A) The diameters of LNCaP and RWPE-2 cell aggregates were measured over 14 days; mean +/- SD, n = 50 from *p<0.05, a paired students t-test was used to show significant differences in diameter at day 7 and 14. (B) FDA/PI stain was used to stain viable cells green and dead cells red on the days shown and the percentage area of dead cells per microaggregate has been quantified to show that the non-cancer prostate cells (RWPE-1) have greater cell death (percentage red pixels) in the microwell inserts compared to the prostate cancer cells (LNCaP and RWPE-2); mean +/- SD, n = 10. A paired students t-test was used to calculate significance*p<0.05. (C) The confocal images and phase contrast images (Day 14 Phase) show that prostate cancer cell lines (RWPE-2 and LNCaP) grow as compact smooth microaggregates until day 14. Conversely, the non-cancer cells (RWPE-1) form dispersed loose clusters which are non-viable and with no definable diameter at day 14. Scale bar is 100 µm.

The purpose of this work was to produce a high-throughput PDMS microaggregate system for the testing of therapeutic drugs for prostate cancer. Initially, we sought to characterize the growth of prostate cancer cells in the microwell system and compare the morphology, and growth rate to normal cell counterparts. PDMS is an inert polymer that is increasingly being used in tissue culture applications [20]. It is suitable because it is inexpensive and compatible with rapid fabrication methodology such as soft lithography [21]. PDMS surfaces have been molded and utilized in culture applications to control the shape and size of single cells [22], and specifically microwells formed from PDMS have been used to manufacture cell aggregates to enhance the differentiation of human embryonic stem cells into neuronal cells [23], [24] and mesenchymal stem cells into osteoblasts or chondrocytes [13], [24], [25]. This study is the first to culture prostate cells on a PDMS microwell surface to controlled aggregate size. Prostate cancer cells were cultured as 3D aggregates of strictly controlled dimensions using a PDMS micro-surface comprised of 600 micro-wells/cm^2^, each being 360 µm by 360 µm (**Figure 1A**). The microwell surface was modified with either 5% pluronic or multi-layered (**Figures 1B and C**). Both surface coatings blocked surface cell adhesion and promoted microaggregate formation. Aggregates formed from LNCaP cells remained viable and increased in metabolism over 7 days at rates comparable to cells grown in 2D culture (**Figures 1B and C**). At day one, after 50,000 cells were seeded, LNCaP cells formed uniform microaggregates of 70 µm diameter (**Figure 2A**). Over a 7 day culture the aggregates uniformly grew to be 120 µm diameter. This appeared to be a limiting diameter and the size did not substantially increase with further culture (**Figure 2A**). RWPE-2 cells formed significantly smaller aggregates of 60 µm at day one, and the microaggregates did not increase in diameter over 14 days (**Figure 2A**). The diameter of the RWPE-1 cells was not measured as the aggregates were unstable, disassociating into loose clusters of cells after three days in culture. RWPE-1 and RWPE-2 cells were cultured in RPMI 5% FBS+PS instead of their recommended growth medium, Keratinocyte-SFM, in order to optimize microaggregate formation. In the latter medium the RWPE-1 and RWPE-2 cells formed dispersed clusters of cells with a high proportion of dead cells after 7 days (**Figure S1A**). Alteration of the growth medium had no significant adverse effects on the cell metabolism compared to the recommended medium (**Figure S1B**). Therefore, RPMI 1640, 5% FBS+P/S was used in all further experimentation.

### Prostate cancer cells are viable in the PDMS microwell system and have a lower proliferation rate relative to monolayer

**Figure 3:**
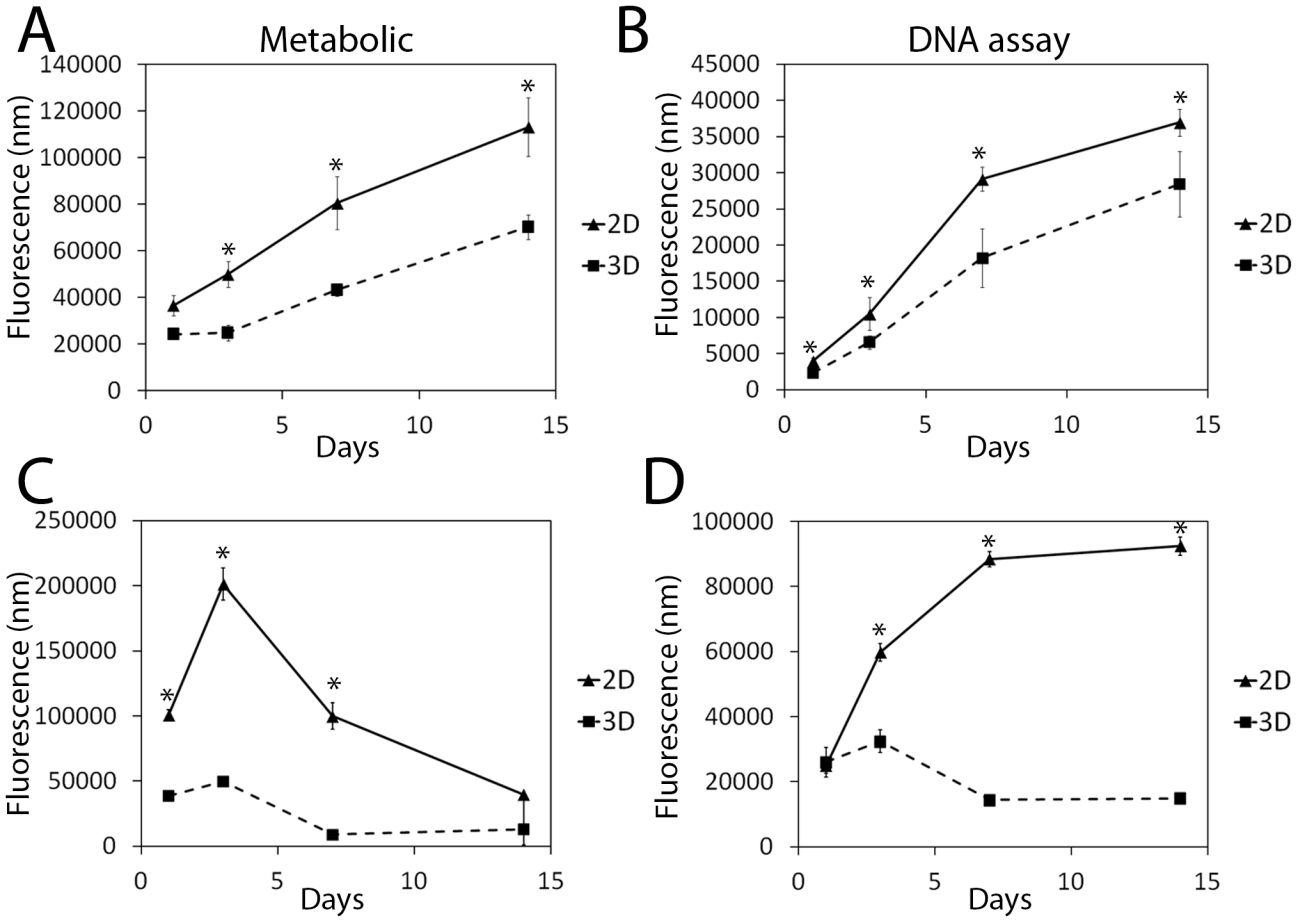
LNCaP microaggregates are metabolically active and proliferative over 14 days; whereas RWPE-2 microaggregates are metabolically dormant and non-proliferative. The viability of LNCaP (A and B) and RWPE-2 (C and D) microaggreagtes versus the same number of cells cultured in 2D was assessed via Alamar blue (A and C) and PicoGreen assay (B and D). LNCaP microaggregates demonstrate an increase in metabolism over 14 days (A) and an increase in DNA content (B). However, RWPE-2 microaggregates show a decrease in metabolism (C) and a decrease in DNA content (D). Mean +/- SD; n = 4. Data represents three experiments. *p<0.05, a paired students t-test was used to determine significance between 2D and 3D.

The viability of the prostate microaggregates was assessed over 14 days by calculating the percentage of dead cells with respect to the live cells (**Figure 2B**) from FDA/PI stained microaggregate confocal images (**Figure 2C**). The FDA dye results show that the prostate cancer cell types, LNCaP and RWPE-2, have an intact cell membrane over 14 days. In contrast, the non-cancerous cell line, RWPE-1 shows a proportional increase in lysed cell membranes over 14 days and display loose organization (day 14 phase). Correspondingly, LNCaP microaggregates demonstrate an increase in metabolism over 14 days (**Figure 3A**) and an increase in DNA content (**Figure 3B**). However, RWPE-2 microaggregates show a clear decrease in metabolism (**Figure 3C**) and a decrease in DNA content (**Figure 3D**) despite having intact cellular membranes according to the FDA staining (**Figure 2B**). Therefore, the RWPE-2 cells are likely to be in a dormant cell state, without cell division or cell death. This is supported by the microaggregate sizing data, which shows that RWPE-2 microaggregates did not significantly increase in diameter (**Figure 2A**). For the RWPE-2 cell 2D data, there was a weak correlation between the assays, despite the clear correlation between the assays for the 3D data; in 2D the metabolism declined after day 3 (**Figure 3C**), however the DNA content continued to increase until day 14 (**Figure 3D**). This indicates that the cells were still able to divide and were therefore not nutrient starved, but shows poor correlation between these assays, which has been previously documented [26]. Nonetheless, the LNCaP PicoGreen data did correlate well with the Alamar blue metabolic data (**Figure 3A and B**). Overall, the metabolism and proliferation of all cells was lower in 3D compared to monolayer (**Figure 3**), which is consistent with previous studies [9]. The 2D surface provides a large surface area for attachment, which is a pre-requisite for successful cell division i.e. focal adhesion anchor points are provided for the dividing cells, which are necessary for proliferation [27]. However, this gives a false representation of the division rate in a tissue and the lower 3D replication rate is more analogous to proliferation rates in tumors.

### Prostate cell lines grown as microaggregates display poor polarity similar to tumors

**Figure 4:**
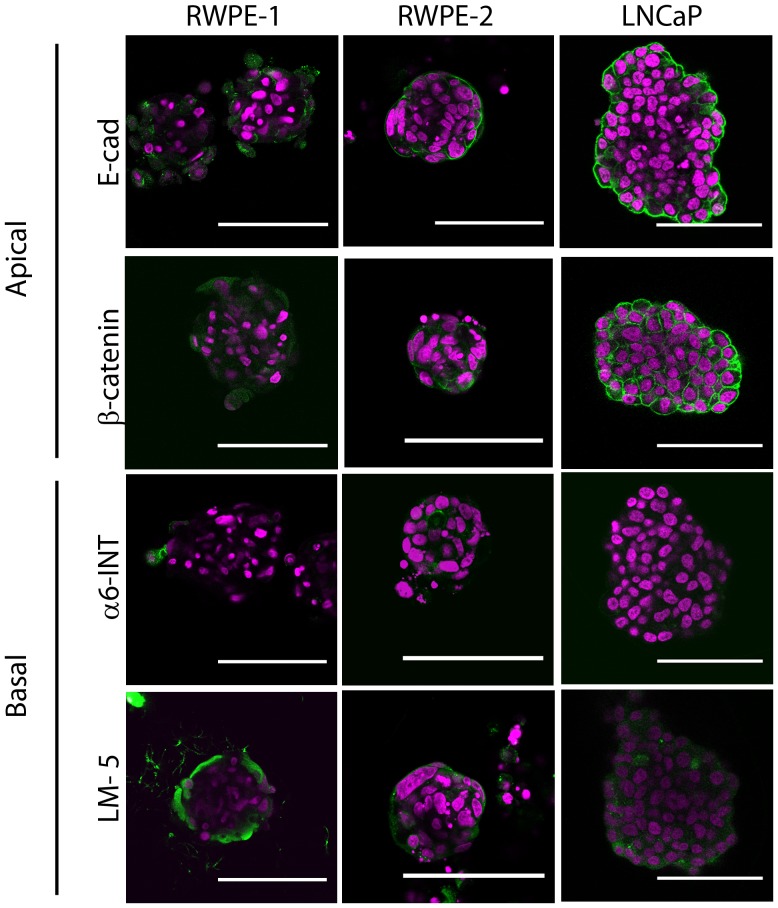
The microaggregates lack polarity as would be observed in a tumor. Microaggregates were fixed at day 7 and immunostained for apical and basal polarity markers (green). E-Cadherin (E-Cad) and β-catenin were used as apical markers and the basal markers were α6-integrin (α6-INT) and Laminin 5 (LM5). Nuclei were stained with DAPI (magenta) and the scale bar is 100 µm.

Microaggregates were fixed at day 7 and immunostained for apical (E-Cadherin and β-catenin) and basal (α6-integrin and laminin-5) markers of 3D polarity (**Figure 4**). These markers were selected according to their previous use as apical and basal surface markers in 3D matrix systems [18], [28]–[30]. From this data, it is clear that all microaggregates did not have a hollow lumen and did not form polar structures, with only Laminin-5 being observed in a position expected for a polar aggregate, which was on the basal surface on the outer edge of the microaggregate (**Figure 4** LM-5****). In order to check that the antibodies were working cell lines cultured in monolayer were also immunostained for the same polarity markers and imaged using confocal Z-sectioning (**Figure S2**). As expected, Laminin 5 and α6-integrin stain the basal surface of all cell types grown in monolayer, with some cytoplasmic staining (**Figure S2B black arrows**). and E-Cad and β-catenin are predominantly expressed at the cell to cell junctions (**Figure S2B white arrows**). Polar 3D microaggregates or spheroids are defined by an apical surface that surrounds a hollow lumen and a basal surface that would attach to the surrounding matrix [18]. The microwell system is devoid of matrix, apart from any cell secreted soluble matrix proteins and there is no scaffold for attachment and hence lack of a clear basal surface. Therefore, it is predictable that the microaggregates lack a hollow lumen and thus an apical surface as matrix tension contributes to tissue organization [31], however some degree of cell organization exsists. For example, LNCaP, RWPE-1 and 2 cells express Laminin-5 on the outer aggregate surface, but do not express α6-integrin. In addition, E-cadherin and β-catenin were found expressed between the cell adhesions of LNCaP and RWPE-2 microaggregates (**Figure 4**), but there is no luminal surface and hence there is no expression of these markers at an apical surface. Thus, the cells are able to express these phenotypic markers but require other cues, likely from an extracellular matrix or surrounding cells such as would be present in the tumor microenvironment, to form organized tissue acini [32]. Although there are no clear differences between the cancer and non-cancer cells lines, the phenotype observed is more indicative of the de-differentiated cancer cell observed in high grade prostate cancer indicating that the assay is still suitable for high throughput drug testing of prostate cancer cells. However, this system is not viable for the culture of non-neoplastic cell lines due to the absence of matrix. But with the addition of Matrigel to the PDMS microwell system, the normal prostate cell line, RWPE-1, formed aggregates that displayed polarity (**Figure S3A and S3B**). With the addition of 8% Matrigel the RWPE-1 aggregates displayed polar organization with the basal expression of α6-integrin (**Figure S3B**) and this polarity was maintained with 1% Matrigel (**Figure S3A**). However, the microaggregates grown in PDMS inserts with Matrigel were larger compared to those grown on inserts alone (**Figure S3C**) or in 8% Matrigel alone (**Figure S3D**). Therefore, the addition of Matrigel provides a matrix to induce α6-integrin polarity, which was lost in RWPE-1 cells cultured on modified PDMS alone. Alpha-6-integrin has been shown to be important for acinus formation in RWPE-1 cells [33], thence the lack of α6-integrin may contribute to the poor microaggregate formation displayed by normal RWPE-1 cells.

### The PDMS microwell system can be used to control apoptotic core

**Figure 5:**
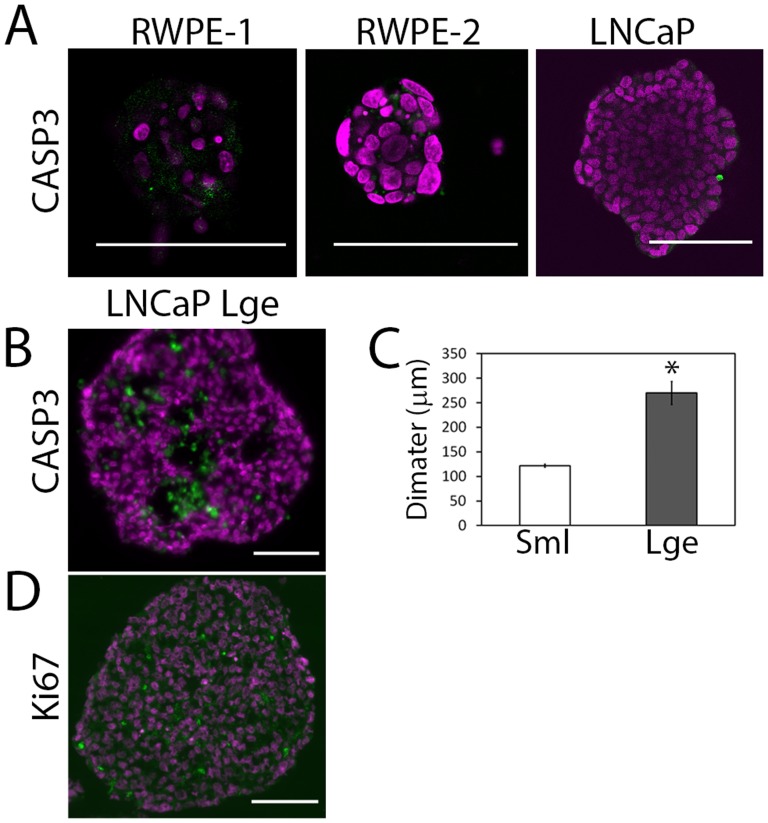
Apoptotic core can be controlled for using differently sized microwells. (A) Cleaved caspase-3 (CASP3; green), an apoptosis marker, was used to stain sections of RWPE-1, RWPE-2 and LNCaP microaggregates. (B) Due to the absence of cleaved caspase-3 in the small LNCaP aggregates, a large microwell (800 µm ×800 µm ×800 µm) was used to create large LNCaP microaggregates with an apoptotic core (CASP3 Lge). (C) The diameter of the LNCaP microaggregates (Sml) was compared with LNCaP microaggregates grown in the large microwells (Lge). A minimum of 50 aggregates were measured per condition. (D) Ki67 (green) was also used to stain proliferating cells within the large LNCaP aggregate. Nuclei were stained with DAPI (magenta); scale bar is 100 µm.

LNCaP cells cultured in the microwell system (360×360 µm), which reach a maximum size of 120 µm do not form an apoptotic core as identified by cleaved caspase-3 staining at day 7 (**Figure 5A**). However, apoptotic core can be controlled for by increasing the size of the micro-well to 800 µm and increasing the seeding cell number to 2000 cells per aggregate. This created aggregates of ∼300 µm in diameter over 7 days with a central core of cleaved caspase-3 staining (**Figure 5B**). An apoptotic core is usually observed in 3D cultures of larger than 500 to 600 µm diameter [34]–[36] however apoptotic cores have been observed even in LNCaP cell aggregates of 200 µm diameter [37]. The variation in observations reflect the fact that effective oxygen delivery is a function of many variables including total culture cell number, and height of the medium as well as aggregate diameter [38]. In our studies we inoculated the larger diameter aggregates with 6 fold greater cell numbers than the smaller aggregates, and because of the cubic relationship in the volume radius equation (V = 4/3πr^3^) this resulted in just less than a doubling of the aggregate diameter (**Figure 5C**). Proliferating cells have previously been shown to be concentrated at the periphery of LNCaP aggregates of 200 µm grown using the agar liquid overlay technique [36]. In our study we used Ki67 staining to reveal that proliferating cells could be found evenly throughout the aggregate and that these cells were not concentrated in any one particular area (**Figure 5D**), perhaps due to the absence of a basal surface provided by a matrix surface. Therefore, we have demonstrated that we can control for apoptotic core using differently sized microwells and that proliferation is evenly distributed throughout the microaggregates.

### Culture of prostate cancer cells as 3D microaggregates increases drug resistance, which is attributed to differences in proliferation

**Figure 6:**
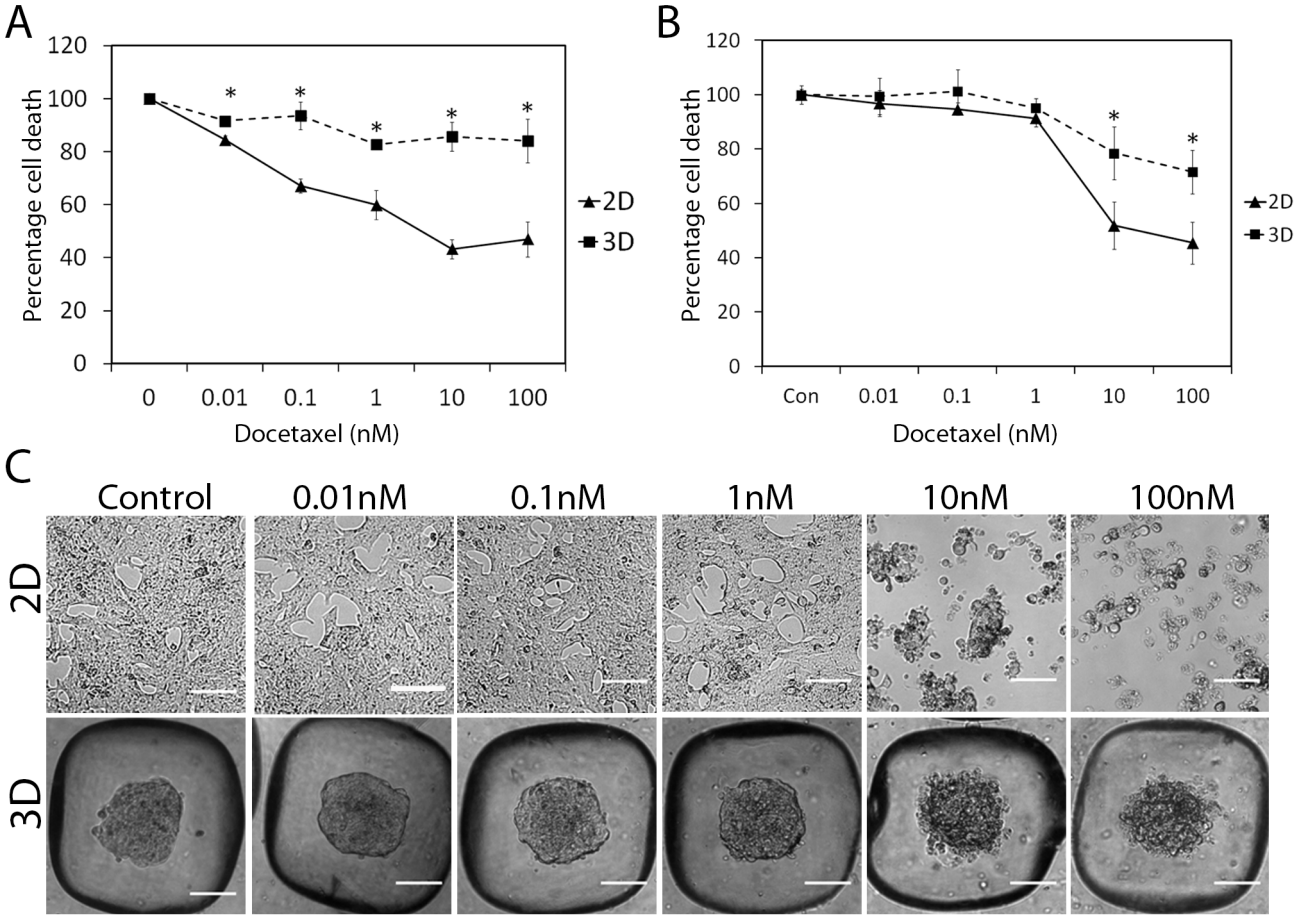
LNCaP microaggregates are more resistant to docetaxel compared to cells grown as monolayers. LNCaP cells (50,000 cells/well) were treated with docetaxel over 48 hrs (A) or 72 hrs (B) at day 2 of growth either in 2D and 3D. Alamar blue was used to assess cell viability. Mean +/- SE, n = 4 biological replicates *P<0.05; the data shown is representative of three independent experiments. (C) Phase contrast images show the effects of docetaxel after 72 hrs on the morphology of the microaggregates (3D) compared to cells grown in monolayer (2D).

LNCaP cells were cultured in 2D or the PDMS microwells for 2 days prior to drug treatment. Up to 1000 nM of docetaxel was added and compared to a DMSO vehicle control. Docetaxel was used as a representative cancer drug in this study due to its routine use in prostate cancer treatment [39]. It prevents mitotic spindle assembly and mitotic cell division, leading to apoptosis, by decreasing the amount of free tubulin needed for microtubule formation [39]. Cells cultured in 3D were more resistant to all doses of docetaxel than those cultured in 2D after 48 hrs (**Figure 6A**). At 72 hrs differences were only observed at the high drug doses of 10 and 100 nM docetaxel (**Figure 6B**). With a longer drug incubation time the IC50 increased for both 2D and 3D. For example in 2D at 48 hrs the IC50 was 11 nM and this increased to 51.3 nM over 72 hrs. In 3D the IC50 was exceptionally high indicating that the 3D microaggregates are highly resistant to docetaxel, the calculated IC50 at 48 hrs was 4.3×10^8^ and 426 nM at 72 hrs. With increasing doses of docetaxel the cells in 3D retained their aggregation, but became more loosely associated, but in 2D the cells detached (**Figure 6C**). It has been reported for several types of cancer that 3D cultures show more resistance to chemotherapeutics [9], [40] and that in 3D, LNCaP cells are more resistant to docetaxel than their monolayer counterparts [9]. In addition, we have shown that LNCaP cells grown in 3D have lower replication compared to their 2D counterparts (**Figure 3B**) and are thus more resistant to docetaxel, which targets proliferating cells [41]. We have shown that these effects are temporary and that when 7 day old microaggregates are returned to a 2D plastic substrate as a single cell suspension they exhibit the same dose response profile as cells cultured in 2D (**Figure S4**). This drug penetration in 3D-systems is slower compared to a single monolayer of cells, which makes it more comparable to solid tumors [42]. Indeed drug penetration into solid tumors is typically 5- to 10-fold slower than in monolayer cultures [43].

### Summary

In summary, the PDMS system can be used to regulate the dimensions of cancer cell microaggregates to replicate different stages of tumor growth. The microwell system produces prostate cancer microaggregates of strictly controlled dimensions using a PDMS micro-surface comprised of 600 micro-wells/cm^2^, each being 360 µm by 360 µm. Surface modification with either 5% pluronic or multi-layering blocks protein adsorption and cell attachment onto the PDMS, and this promotes microaggregate formation. LNCaP aggregates were viable and the cell number in the aggregates increased over time. Apoptotic core in LNCaP cells aggregates can be controlled by using either small (360×360 µm) or large (800×800 µm) dimension microwells. The cancer microaggregates, formed from LNCaP and RWPE-2 cells lack polarity as would be observed in a tumor while the normal RWPE-1 cells form a basal surface as identified by α6-integrin expression after the addition of Matrigel matrix to the microwell plates. The normal prostate cells, RWPE-1, cultured without matrix were not viable in the system and 80% of the cells in 3D cultures had died by day 14. This system can be used for high-throughput drug testing for cancer cells in 3D. The small and large microwell surfaces enable manufacture of 600 or 150 microaggregates per cm^2^, respectively. A 96 well plate with the small microwell surface enables the uniform manufacture ∼34,000 microaggregates, and this could be integrated with fluidics robotics to enable high throughput drug testing. Using this system, we have shown that LNCaP microaggregates are more resistant to docetaxel compared to cells grown as monolayers and that when the microaggregates are dispersed and returned to monolayer, they exhibit the same dose response to docetaxel as those cells cultured in monolayer. Future advances in the use of this technology include the addition of stromal cell types including fibroblasts, mesenchymal stromal cells, endothelium and osteoblasts to better mimic the tumor microenvironment.

## Supporting Information

Figure S1 The viability of RWPE-1 and RWPE-2 cells cultured in keratincyte-SFM medium. (A) RWPE-1 and RWPE-2 cells were grown in Keratinocyte-SFM+BPE+EGF+FGF (KSFM) over 7 days to observe the morphology and viability of the microaggregates. The viability of RWPE-1 and RWPE-2 cells was determined using FDA/PI staining and imaged using confocal microscopy (upper panel) and phase contrast microscopy (Ph; lower panel). Scale bar is 100 µm. (B) WST-1 cell proliferation assay was used to determine metabolism of RWPE-1 and RWPE-2 cells grown in different medium compositions. KSFM was compared to RPMI 1640+5% FBS (labeled as R5%), and a 1∶1 ratio mix of both media types. RPMI without serum (labeled as R) was used as a negative control. Mean +/-SD; n = 4 *P<0.05.
(TIF)

Figure S2 Markers of spheroid polarity in monolayer. (A) Monolayers of RWPE-1, RWPE-2 or LNCaP cells were fixed and immunostained for apical (E-Cadherin/E-Cad and β-catenin) and basal polarity markers (α6-integrin/α6-INT and laminin-5/LM-5) (green). (B) Shows the confocal Z-projection of each of the stained images. LM-5 and α6-INT stain the basal surface of all cell types (black arrow heads), and LNCaP cells express LM-5 throughout the cell cytoplasm. E-Cad and β-catenin are predominantly expressed at the cell to cell junctions (white arrow heads) and on the apical surface of all cell types. However, some basal staining does occur. In all images nuclei are stained with DAPI (blue) and F-actin was stained with Phalloidin (red). The scale bar is 100 µm.
(TIF)

Figure S3 3D culture of RWPE-1 cells in Matrigel basement membrane matrix. RWPE-1 cells were cultured in either (A) 1% or (B) 8% Matrigel (BD Biosciences) in the presence of Keratinocyte-SFM medium supplemented with 2% BPE (Gibco) for 7 days. The polarity of aggregates formed from microwell inserts alone (C) or Matrigel alone (D) was compared. Micro-aggregates were immunostained with α6-integrin (green), F-actin (red) and DAPI (blue). The panel in D shows the magnified edge of the microaggregate with Matrigel alone.
(TIF)

Figure S4 LNCaP cells grown as 3D microaggregates and returned to tissue culture plastic respond the same way to docetaxel as monolayer cells. LNCaP cells were seeded in 48-well plates with PDMS inserts and cultured for 7 days prior to being typsinized and re-plated in a 48-well plate without PDMS insert. Fresh LNCaP cells, which had not been subjected to 3D culture were plated at the same time. Two days post-seed cells were treated with 0.01-1000 nM docetaxel (Sigma-Aldrich) for 72 h and viability read using Alamar blue assay. There was no significant difference between the cells cultured in monolayer (2D) and those that had previously been cultured as 3D microaggregates (3D RS) n = 3, *p<0.05.
(TIF)
