# Correction: 3D Cultures of Prostate Cancer Cells Cultured in a Novel High-Throughput Culture Platform Are More Resistant to Chemotherapeutics Compared to Cells Cultured in Monolayer

**DOI:** 10.1371/journal.pone.0125641

**Published:** 2015-04-22

**Authors:** Karen F. Chambers, Eman M. O. Mosaad, Pamela J. Russell, Judith A. Clements, Michael R. Doran

There is an error in Fig 5C. The y-axis should be labeled “Diameter (μm).” Please see the corrected Fig 5 here.

**Fig 5:**
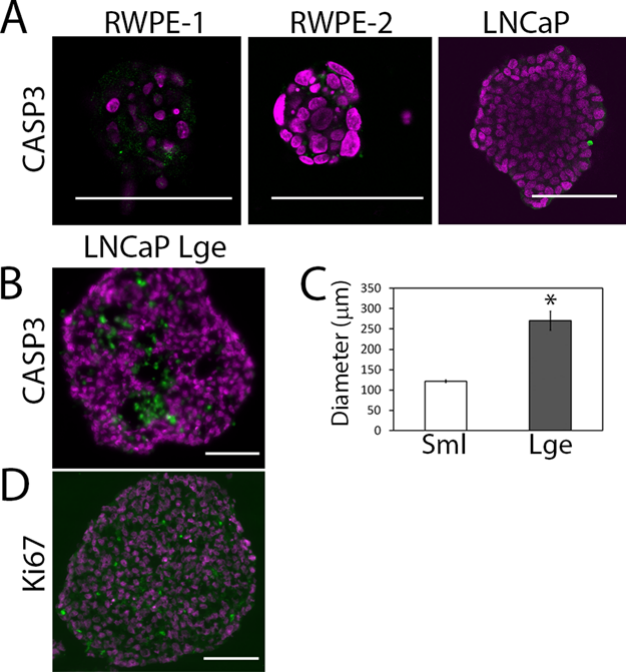
Apoptotic core can be controlled for using differently sized microwells. (A) Cleaved caspase-3 (CASP3; green), an apoptosis marker, was used to stain sections of RWPE-1, RWPE-2 and LNCaP microaggregates. (B) Due to the absence of cleaved caspase-3 in the small LNCaP aggregates, a large microwell (800 μm ×800 μm ×800 μm) was used to create large LNCaP microaggregates with an apoptotic core (CASP3 Lge). (C) The diameter of the LNCaP microaggregates (Sml) was compared with LNCaP microaggregates grown in the large microwells (Lge). A minimum of 50 aggregates were measured per condition. (D) Ki67 (green) was also used to stain proliferating cells within the large LNCaP aggregate. Nuclei were stained with DAPI (magenta); scale bar is 100 μm.

There is an error in Fig 6A and 6B. The y-axis should be labeled “percent cell viability.” Please see the corrected Fig 6 here.

**Fig 6:**
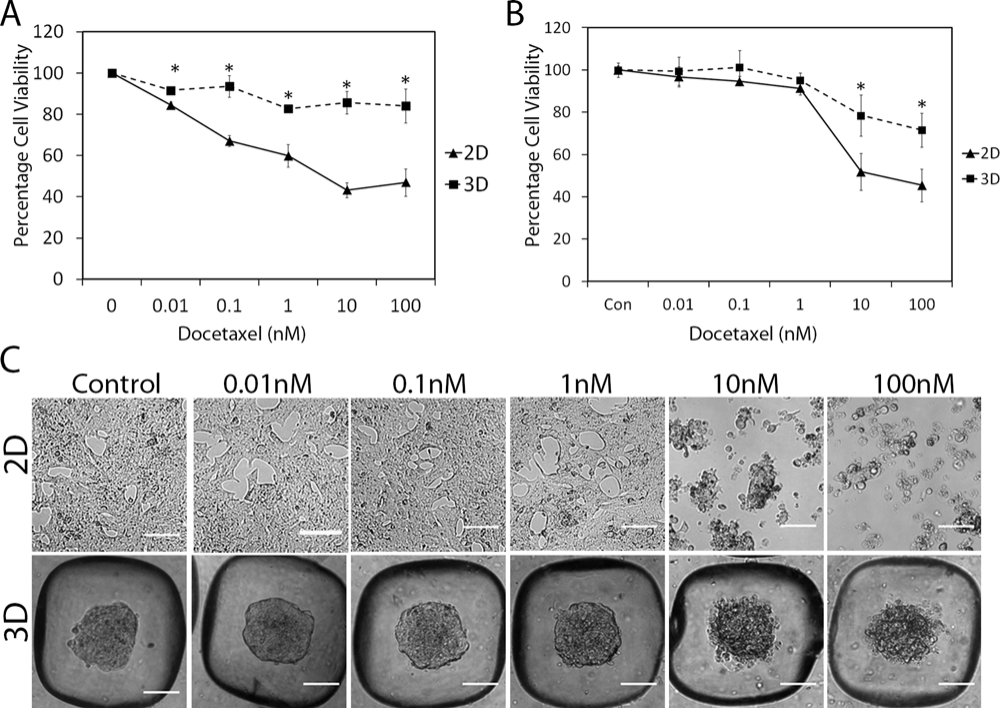
LNCaP microaggregates are more resistant to docetaxel compared to cells grown as monolayers. LNCaP cells (50,000 cells/well) were treated with docetaxel over 48 hrs (A) or 72 hrs (B) at day 2 of growth either in 2D and 3D. Alamar blue was used to assess cell viability. Mean +/- SE, n = 4 biological replicates *P<0.05; the data shown is representative of three independent experiments. (C) Phase contrast images show the effects of docetaxel after 72 hrs on the morphology of the microaggregates (3D) compared to cells grown in monolayer (2D).
