# Supplementary figures and images for: 3D Cultures of Prostate Cancer Cells Cultured in a Novel High-Throughput Culture Platform Are More Resistant to Chemotherapeutics Compared to Cells Cultured in Monolayer
Source: PLoS One. 2014 Nov 7;9(11):e111029. doi: 10.1371/journal.pone.0111029 (PMC4224379; doi:10.1371/journal.pone.0111029)

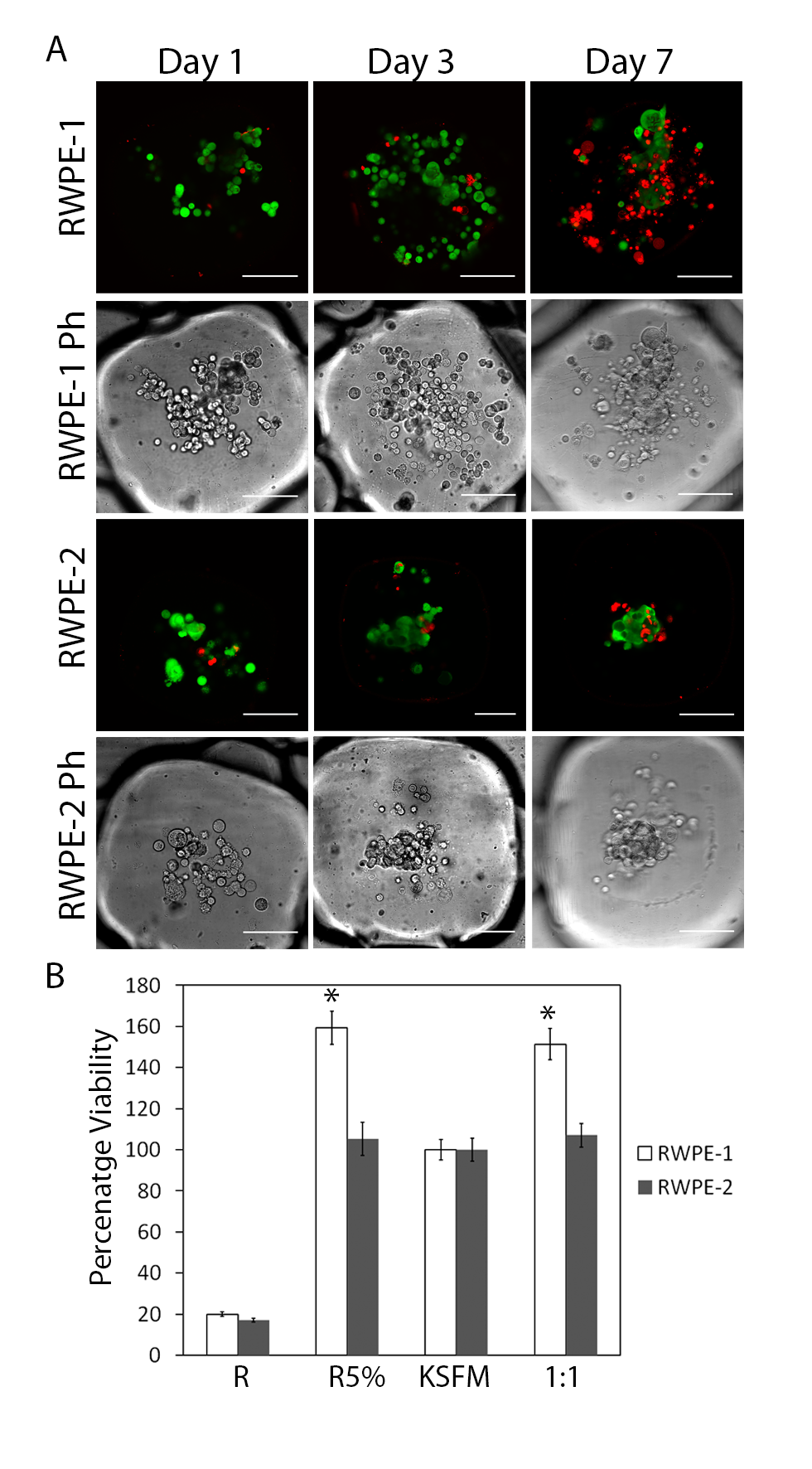

Supplement: Figure S1 — The viability of RWPE-1 and RWPE-2 cells cultured in keratincyte-SFM medium. (A) RWPE-1 and RWPE-2 cells were grown in Keratinocyte-SFM+BPE+EGF+FGF (KSFM) over 7 days to observe the morphology and viability of the microaggregates. The viability of RWPE-1 and RWPE-2 cells was determined using FDA/PI staining and imaged using confocal microscopy (upper panel) and phase contrast microscopy (Ph; lower panel). Scale bar is 100 µm. (B) WST-1 cell proliferation assay was used to determine metabolism of RWPE-1 and RWPE-2 cells grown in different medium compositions. KSFM was compared to RPMI 1640+5% FBS (labeled as R5%), and a 1∶1 ratio mix of both media types. RPMI without serum (labeled as R) was used as a negative control. Mean +/-SD; n = 4 *P<0.05. (TIF) [file pone.0111029.s001.tif]

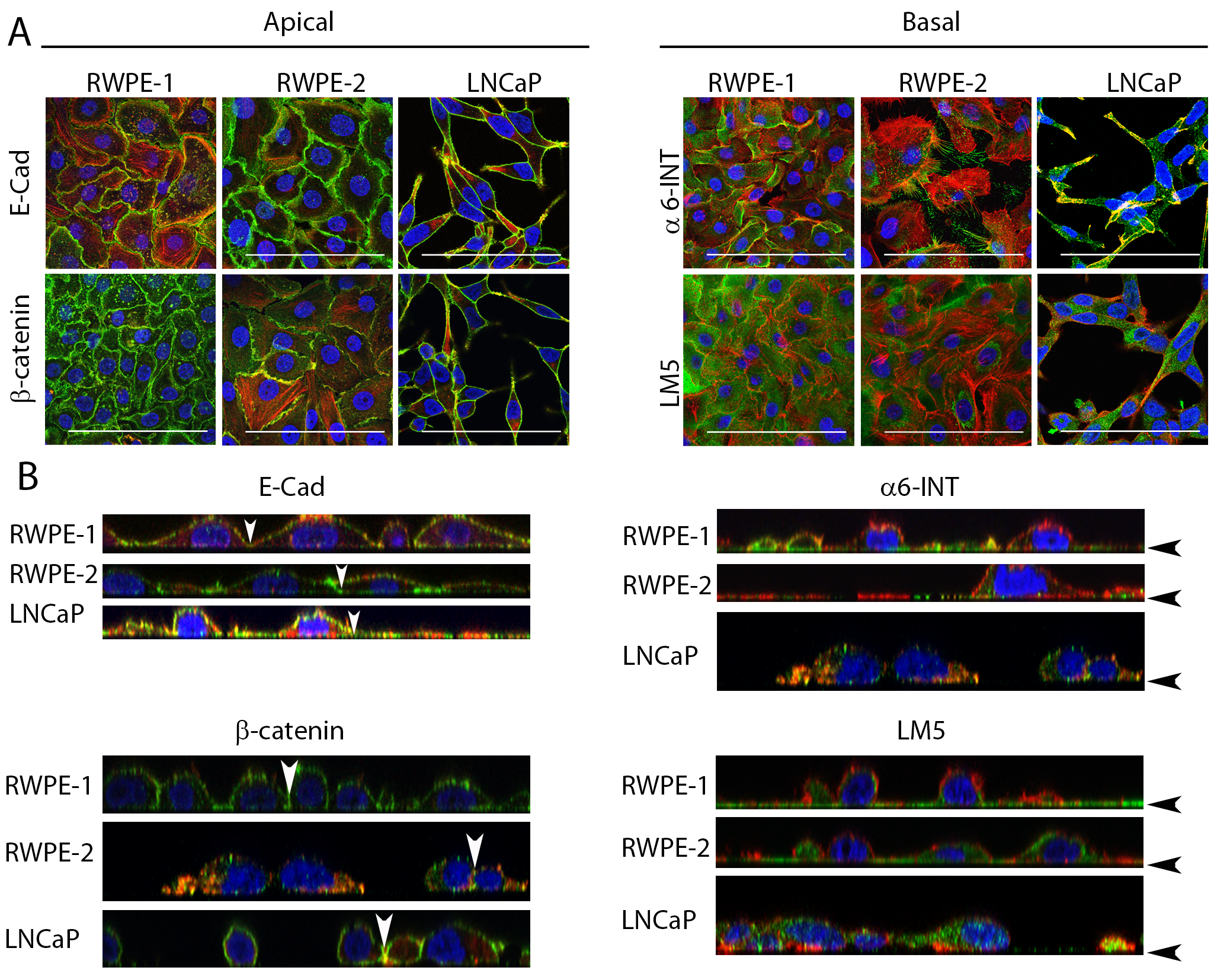

Supplement: Figure S2 — Markers of spheroid polarity in monolayer. (A) Monolayers of RWPE-1, RWPE-2 or LNCaP cells were fixed and immunostained for apical (E-Cadherin/E-Cad and β-catenin) and basal polarity markers (α6-integrin/α6-INT and laminin-5/LM-5) (green). (B) Shows the confocal Z-projection of each of the stained images. LM-5 and α6-INT stain the basal surface of all cell types (black arrow heads), and LNCaP cells express LM-5 throughout the cell cytoplasm. E-Cad and β-catenin are predominantly expressed at the cell to cell junctions (white arrow heads) and on the apical surface of all cell types. However, some basal staining does occur. In all images nuclei are stained with DAPI (blue) and F-actin was stained with Phalloidin (red). The scale bar is 100 µm. (TIF) [file pone.0111029.s002.tif]

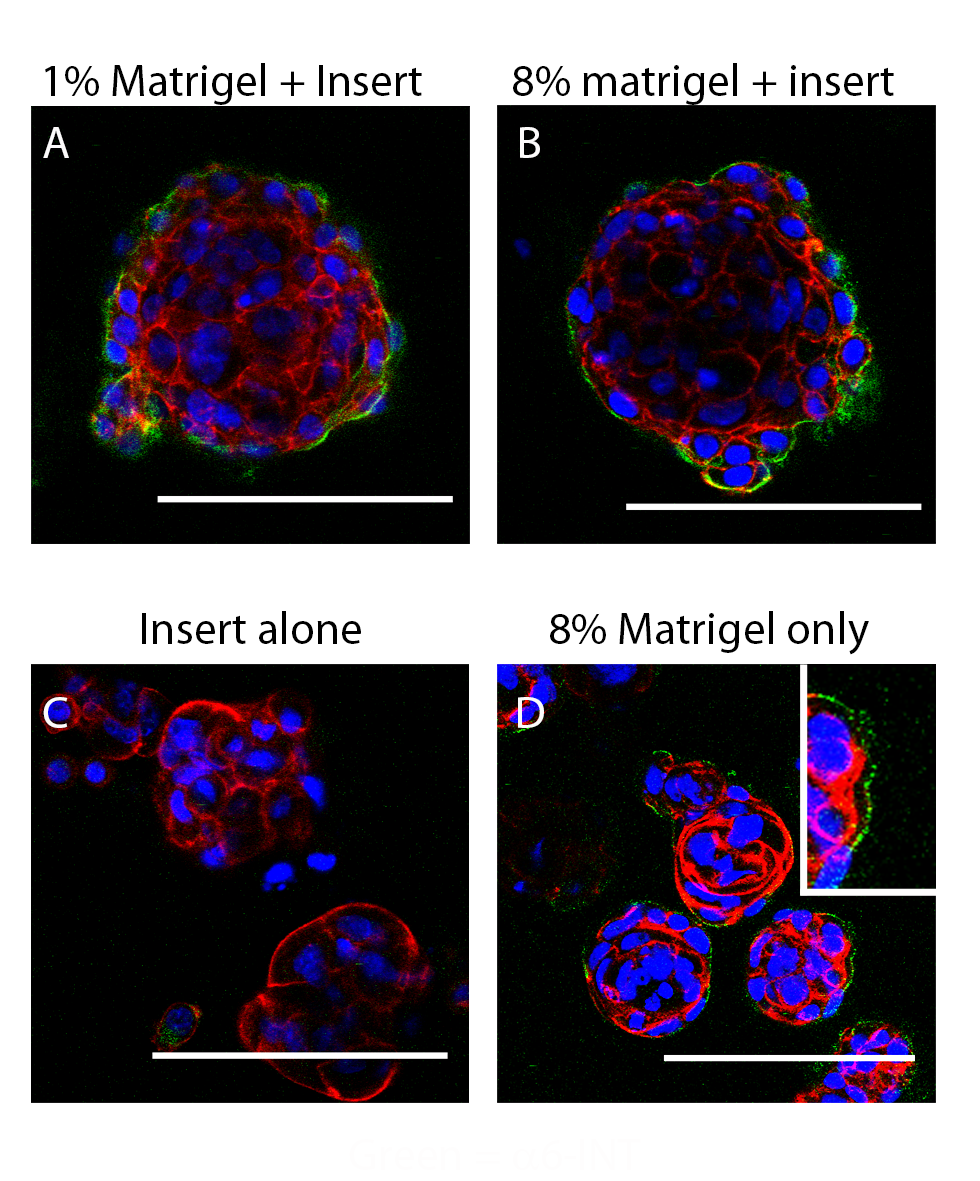

Supplement: Figure S3 — 3D culture of RWPE-1 cells in Matrigel basement membrane matrix. RWPE-1 cells were cultured in either (A) 1% or (B) 8% Matrigel (BD Biosciences) in the presence of Keratinocyte-SFM medium supplemented with 2% BPE (Gibco) for 7 days. The polarity of aggregates formed from microwell inserts alone (C) or Matrigel alone (D) was compared. Micro-aggregates were immunostained with α6-integrin (green), F-actin (red) and DAPI (blue). The panel in D shows the magnified edge of the microaggregate with Matrigel alone. (TIF) [file pone.0111029.s003.tif]

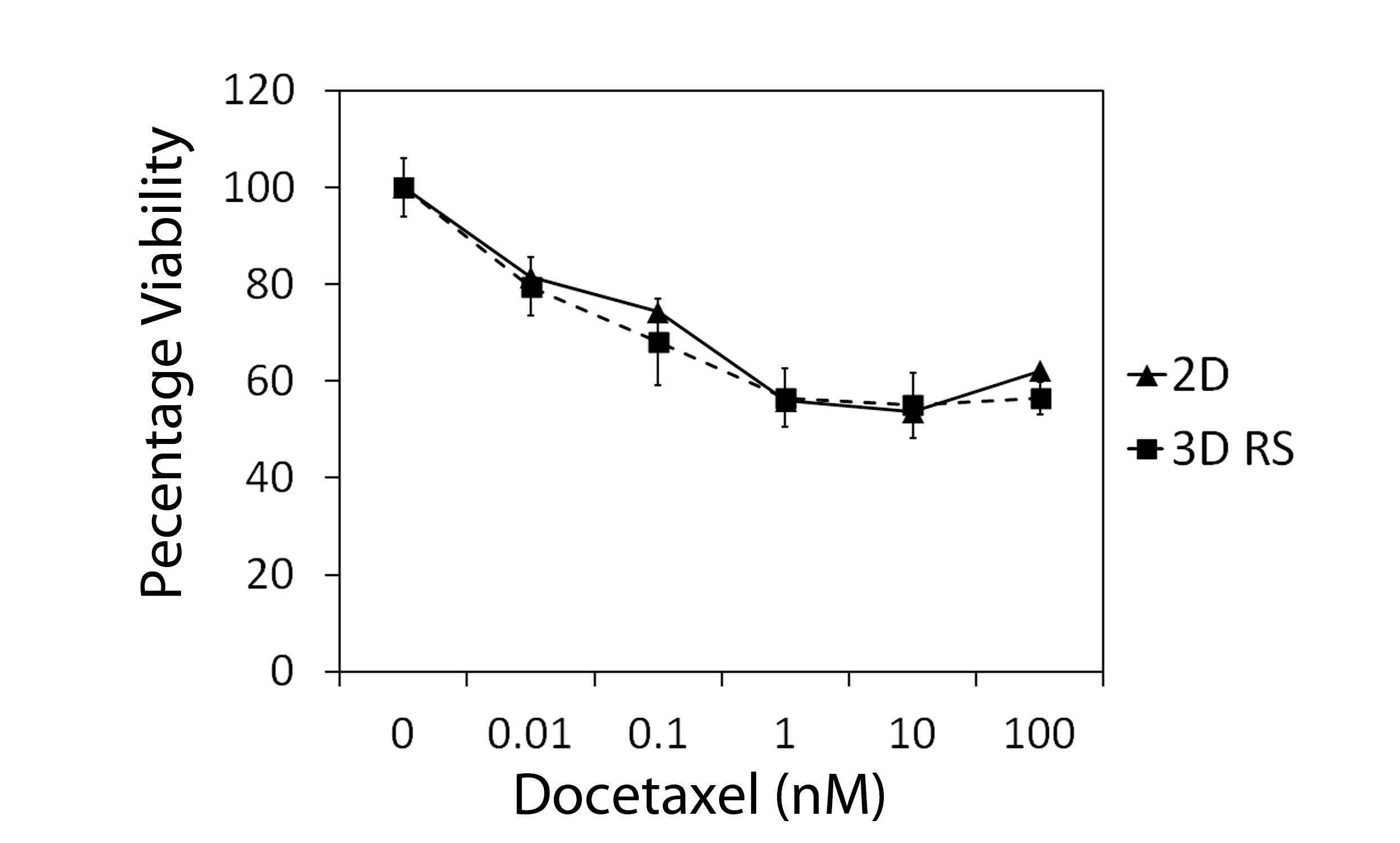

Supplement: Figure S4 — LNCaP cells grown as 3D microaggregates and returned to tissue culture plastic respond the same way to docetaxel as monolayer cells. LNCaP cells were seeded in 48-well plates with PDMS inserts and cultured for 7 days prior to being typsinized and re-plated in a 48-well plate without PDMS insert. Fresh LNCaP cells, which had not been subjected to 3D culture were plated at the same time. Two days post-seed cells were treated with 0.01-1000 nM docetaxel (Sigma-Aldrich) for 72 h and viability read using Alamar blue assay. There was no significant difference between the cells cultured in monolayer (2D) and those that had previously been cultured as 3D microaggregates (3D RS) n = 3, *p<0.05. (TIF) [file pone.0111029.s004.tif]
